# Cerebral pulsatility might be a driver of neurofunctional reorganization in the aging brain: an MRI and NIRS study

**DOI:** 10.3389/fnagi.2025.1486775

**Published:** 2025-05-20

**Authors:** Hanieh Mohammadi, Parikshat Sirpal, Louis Bherer, Frédéric Lesage, Yves Joanette

**Affiliations:** ^1^Montreal Heart Institute, Montreal, QC, Canada; ^2^Faculty of Medicine, University of Montreal, Montreal, QC, Canada; ^3^Laboratory of Optical and Molecular Imaging, Biomedical Engineering Institute, Polytechnique Montreal, Montreal, QC, Canada; ^4^School of Electrical and Computer Engineering, Gallogly College of Engineering, University of Oklahoma, Norman, OK, United States; ^5^University Institute of Geriatrics of Montreal (CRIUGM), Montreal, QC, Canada

**Keywords:** cerebral pulsatility, functional reorganization, aging brain, Stroop task, near-infrared spectroscopy, phase-contrast MRI

## Abstract

Age-related increases in cerebral pulsatility are thought to stress cerebral microcirculation, with effects that may vary across different brain regions. The aging brain also undergoes neurofunctional changes to preserve and, in some cases, enhance cognitive abilities. This study investigated the association between cerebral pulsatility and neurofunctional changes in aging. Sixty healthy adults were divided into two groups of younger (aged 19–31 years) and older adults (aged 62–75 years). Participants underwent near-infrared spectroscopy (NIRS) imaging, including a resting-state recording to capture the baseline cortical cerebral pulsatility index, followed by a Stroop task to assess cortical hemodynamic-evoked activity. Phase-contrast magnetic resonance imaging (PC-MRI) was also performed to measure pulsatility in the large arteries supplying the brain. Results indicated that older adults exhibited a significant interhemispheric difference in cerebral pulsatility index, with a higher index in the left hemisphere compared to the right. In older adults, a greater interhemispheric difference in cerebral pulsatility index was associated with larger task-evoked oxyhemoglobin concentration changes in the right hemisphere and smaller changes in the left hemisphere for the Stroop task. Younger adults, in contrast, showed no significant interhemispheric difference in the cerebral pulsatility index nor a significant correlation with task-evoked activations. These findings suggest that age-related changes in cerebral pulsatility might contribute to or potentially drive functional reorganization in the aging brain. Further investigation is needed to provide more insight into this finding.

## 1 Introduction

Contraction and relaxation of the heart generate pulsatile blood flow and pressure that are transmitted into the brain through the internal carotid arteries (ICAs) and vertebral arteries (VAs), where they are termed ‘cerebral pulsatility’. In youth, elastic arteries smooth pulsatility fluctuations by optimally distending during systole and optimally recoiling during diastole (Mitchell et al., 2011). Consequently, despite the strong pulsatility in the central arteries, there is a relatively continuous blood flow in the cerebral microcirculation (O’Rourke and Hashimoto, 2007), a condition that is required to support neural metabolism and cognitive health (Mitchell et al., 2011; Mitchell, 2018).

Aging is associated with increased arterial stiffness and reduced pulsatility damping, which, among other factors, leads to increased pulsatility reaching the brain (Mitchell et al., 2011). This age-related change exerts higher mechanical stress on the brain microvasculature (Nichols et al., 1991), some of which dissipates into surrounding regions (Mitchell, 2015). As pulsatility travels to the cortex, it is altered by differences in arterial geometry and supply, venous drainage, and varying degrees of vascular wall damage along its path. As a result, pulsatility varies across cortical regions, with some areas experiencing higher levels than others (Kim et al., 2021). These spatial variations raise important questions: Can regional differences in cerebral pulsatility influence neurovascular coupling or alter local hemodynamic responses? Furthermore, can prolonged exposure to increased pulsatility within specific brain regions lead to functional reorganization and shifts in activation patterns?

To the best of our knowledge, the underlying mechanisms of age-related functional reorganization have not been extensively investigated in the literature. However, certain hypotheses have been proposed. For instance, Stern (2009) suggested that as the brain networks that are typically engaged in youth (the initially recruited networks) become less efficient with age, the brain compensates by recruiting additional or alternative networks to support cognitive function. Stern hypothesized that these alternative brain networks may be less affected by age-related changes or pathological damage. In line with this hypothesis, Steffener et al. (2009) reported that as gray matter volume decreases with age, alternative regions, often those with greater structural integrity, are engaged in older adults. This further supports other findings suggesting that cognitive tasks can be executed through multiple neural pathways and that age-related activation shifts reflect flexible neural reallocation to adapt to structural and functional changes (Martins et al., 2015). Therefore, the initially dominant network, once sufficient in youth, may become less efficient with aging, which prompts the engagement of additional or alternative regions to maintain cognitive performance (Ansado et al., 2013; Martins et al., 2015; Reuter-Lorenz and Cappell, 2008). While Stern’s model is centered on age-related changes in neural efficiency and compensatory recruitment (Stern, 2009), we propose that vascular factors—specifically cerebral pulsatility—may underlie or contribute to these neurofunctional changes. In this view, we hypothesize that higher regional cortical pulsatility may impair local neurovascular coupling, thereby impacting local cortical hemodynamic responses. In turn, the aging brain functionally reorganizes and may engage regions that are less affected by age-related pulsatility changes.

Investigating this hypothesis requires evaluating pulsatility at the cortical level, particularly in regions co-localized with task-evoked hemodynamic activations. However, the dense microvascular network with billions of small branches and diverse flow directions (Fabiani et al., 2014) has made these assessments challenging in previous studies. Methods such as pulse wave velocity (PWV), transcranial Doppler ultrasound (TCD), carotid ultrasound, and phase-contrast MRI (PC-MRI) are typically used to measure different aspects of pulsatility (Waldstein et al., 2008; Harris et al., 2018; Chung et al., 2017; Zarrinkoob et al., 2016). These methods are generally applied to larger vessels with a single predominant flow direction and, in conventional use, are not well suited for evaluating pulsatility in cerebral microcirculation. Thus, the majority of previous studies have focused on correlations between pulsatility indices in large central or cerebral arteries and cognitive performance (Chung et al., 2017; Harris et al., 2018; Waldstein et al., 2008) rather than directly investigating cortical microvascular pulsatility in relation to cognitive function.

Near-infrared spectroscopy (NIRS) allows for the assessment of pulsatility in cortical microcirculation by emitting near-infrared light from NIRS sources and detecting the backscattered light that has diffused through the brain’s microcirculation (Themelis et al., 2007). With each heartbeat, fluctuations in local oxygenated blood volume ﻿alter the absorption of near-infrared light (Themelis et al., 2007). Since NIRS has a high temporal resolution, it can capture these rapid changes in light intensity. The recorded data include oscillations resembling heartbeats, in which their amplitude serves as a proxy index for local pulse pressure (Tan et al., 2017). Indeed, studies have quantified pulsatility using various NIRS-derived indices and shown its association with cognitive performance (Tan et al., 2017). A previous study reported a reduction in cerebral pulse amplitude, a NIRS-derived index, following vasodilation induced by voluntary breath-holding (Tan et al., 2016). Although in recent years, blood-oxygen-level-dependent functional magnetic resonance imaging (fMRI) has also been used to extract a proxy index of cerebral pulsatility (Potvin-Jutras et al., 2025; Kim et al., 2021), NIRS offers a valuable alternative for research as it offers a cost-effective and accessible method for the assessment of cerebral pulsatility in naturalistic settings, with primary sensitivity to arterial pulsatility (Mohammadi et al., 2021a).

In the present study, we investigated cerebral pulsatility using an index referred to as “*cerebral pulsatile stress”* (Mohammadi et al., 2025), which builds on the established concept of “pulsatile stress” in peripheral arteries—typically calculated as the product of pulse pressure (representing the mechanical stress on the vascular wall) and heart rate (reflecting the frequency of exposure to this stress cycle) to index cumulative stress load (Jae et al., 2012; Lima et al., 2021). In the present study, this concept was extended to the brain by incorporating cerebral pulse amplitude derived from NIRS data. However, as this index is less explored in the literature in the cerebral context, a PC-MRI-based measure of pulsatility was also incorporated as an established method (Chang et al., 2011). For the cognitive task, the color–word matching Stroop interference task was used, which is a widely recognized cognitive task known to elicit specific neurofunctional activations associated with age-related changes (Zysset et al., 2007).

The primary aim of this study was to explore the impact of aging on the association between cerebral pulsatility index and hemodynamic-evoked activations for the Stroop test. More broadly, this study aimed to shed light on the underlying mechanisms of adaptive brain aging.

## 2 Materials and methods

### 2.1 Study population and screening procedure

Seventy-one participants were enrolled in the study. A physician reviewed the blood test results of the participants, and only those with an eligible clinical profile were included in the study. Of these, 60 participants met the eligibility criteria with sufficient data quality (see Section 2.8) for further analysis. The selected participants were divided into two groups: healthy older adults (62–75 years old) and healthy younger controls (19–31 years old). The demographics of the participants are provided in Table 1.

**Table 1:** Characteristics of the study population.

Characteristics	Healthy younger adults	Healthy older adults
Participants (female/male) (*n*)	16/14	15/15
Age (years)	25.41 (2.97)	68.16 (2.87)
Height (cm)	171.92 (8.47)	167.11 (10.84)
Weight (kg)	68.67 (7.33)	68.30 (14.05)
Resting SBP (mmHg)	122.60 (4.09)	122.60 (4.61)
Resting DBP (mmHg)	80.96 (7.16)	79.93 (7.86)
Pulse pressure (mmHg)	39.11 (5.26)	42.07 (6.16)
Heart rate (beats/min)	70.17 (6.83)	71.46 (5.28)
Blood sample parameters		
Total cholesterol (mmol/L)	5.12 (0.34)	5.62 (0.39)
Cholesterol-HDL (mmol/L)	1.56 (0.28)	1.42 (0.59)
Cholesterol-LDL (mmol/L)	1.97 (0.66)	2.16 (0.61)
Cholesterol non-HDL (mmol/L)	2.41 (0.77)	3.64 (1.09)
Triglycerides (mmol/L)	0.93 (0.57)	1.37 (0.51)
C-reactive protein (mg/L)	1.42 (0.81)	2.79 (2.35)
Sodium (mmol/L)	139.53 (1.57)	140.42 (7.78)
Potassium (mmol/L)	4.09 (0.18)	4.27 (0.25)
Chloride (mmol/L)	104.80 (1.71)	104.76 (2.25)
Medication therapy		
Aspirin, *n* (%)	0 (0)	2 (6)

Variables are presented as mean (standard deviation). *n*, number of participants; %, percentage of participants; SBP, Systolic blood pressure; DBP, Diastolic blood pressure; LDL, Low-density lipoprotein; HDL, High-density lipoprotein.

In the present study, inclusion criteria were healthy, native, French-speaking, MRI-compatible, right-handed adults (as assessed using the Edinburgh Handedness Inventory; Oldfield, 1971). Exclusion criteria included left-handedness, claustrophobia, and any of the following self-reported conditions: diabetes mellitus, hypertension, smoking, epilepsy, asthma, infarction, or neurodegenerative disease (e.g., Alzheimer’s disease, Parkinson’s disease, or multiple sclerosis). Participants were also excluded if they reported using medications that affect the vascular system (e.g., warfarin, heparin, Xarelto), the nervous system (e.g., benzodiazepines, antidepressants), or cardiovascular function (e.g., beta-blockers, calcium channel blockers). The cutoff value for hypertension was defined as a blood pressure of ≥140/90 mmHg. Participants should meet the following blood test criteria: low-density lipoprotein (LDL) ≤ 3.36 mmol/L, high-density lipoprotein (HDL) ≥ 1.03 mmol/L, and C-reactive protein ≤ 3 mg/L. The level of electrolytes should be within the normal range: sodium 133–145 mmol/L, potassium 3.5–5 mmol/L, and chloride 100–109 mmol/L. A physician reviewed all blood test results to confirm participant eligibility. The study protocol was approved by the ethics committees of the Centre de Recherche de l’Institut Universitaire de Gériatrie de Montréal (CRIUGM), the Polytechnique Montréal, and the Montreal Heart Institute. All participants provided written informed consent in accordance with the Declaration of Helsinki.

### 2.2 Data collection sessions

Our multisession study consisted of three visits, each lasting approximately 2 h. Participants were asked to avoid consuming caffeinated beverages on the day of the experiment (Vidyasagar et al., 2013). The first visit included blood work, in which 7 mL of blood was collected from participants following an overnight fasting. The blood draw was conducted as the first task in the morning. As part of the study protocol, participants were instructed to avoid exercising or walking long distances while fasting. After the blood test, participants were given a light breakfast, followed by neuropsychological tests (see Table 2). The first session concluded with familiarizing participants with the NIRS and MRI acquisitions. The second visit began with the measurement of their weight and height and concluded with the acquisition of MRI data. At the third visit, functional NIRS data were collected simultaneously with electrocardiography (ECG). Blood pressure was also measured, and pulse pressure was calculated as the difference between the systolic maximum and diastolic minimum. The time of the experiment was counterbalanced between participants.

**Table 2:** Cognitive characteristics and depression scale for the study population.

Test name	Variable	Healthy younger adults	Healthy older adults	*t*-test
MoCA		-	27.21 (1.58)	-
TMT	TMT-A	20.01 (4.41)	34.04 (11.42)	<0.001
	TMT-B	47.69 (13.18)	80.47 (34.28)	<0.001
WAIS-III	Vocabulary	51.53 (8.96)	53.56 (8.50)	0.34
	Similarities	25 (3.99)	22.72 (5.60)	0.04
	Matrix reasoning	21.53 (3.17)	16.02 (6.32)	<0.001
	Direct digit span	6.76 (0.93)	4.91 (1.13)	<0.001
	Reverse digit span	5.73 (0.73)	4.27 (0.56)	0.004
RAVLT	Immediate recall	10.39 (2.61)	9.43 (2.51)	0.019
	20-min delayed recall	10.90 (1.34)	8.45 (3.25)	<0.001
Stroop	Naming	27.24 (4.73)	29.28 (6.63)	0.014
	Reading	20.34 (5.33)	22.17 (3.88)	0.112
	Inhibition	43.21 (7.82)	60 (13.97)	<0.001
	Switching	47.89 (9.41)	63.46 (13.61)	<0.001
GDS		-	3.59 (3.59)	-
BDI		5.96 (7.05)	-	-

MoCA, Montreal Cognitive Assessment (score); TMT-A/B, trail-making test A/B (time in seconds); WAIS-III, Wechsler Adult Intelligence Scale (score); RAVLT, Rey auditory verbal learning test (number of recalled words); GDS, Geriatric Depression Scale (score); BDI-II, Beck Depression Inventory-II (depression scale); Stroop test, each subsection (time in seconds). Results are presented as means (standard deviation). *p*-values from two-sample *t*-tests indicate differences between older adults and younger controls.

### 2.3 Assessment of cognitive function

The Montreal Cognitive Assessment (MoCA; Nasreddine et al., 2005) and the Geriatric Depression Scale (GDS; Yesavage et al., 1982) were administered to the older participants. The Trail Making Test (TMT-A and TMT-B; Reitan, 1958) was used to evaluate processing speed and executive function. The Beck Depression Inventory-II (BDI-II; Beck et al., 1961) was administered to assess depression in the younger participants. Subtests of the Wechsler Adult Intelligence Scale (WAIS-III; Wechsler, 1997), including vocabulary, similarities, matrix reasoning, forward and backward digit span, and the digit symbol substitution test (DSST), were administered to all participants. Additionally, the Rey Auditory Verbal Learning Test (RAVLT; Rey, 1964) and four subtests of the Stroop test (Stroop, 1935) were administered. Table 2 summarizes the cognitive measures and the two-sample t-test p-values comparing the groups. The cognitive measures of the older adults were within the average range for their age, indicating normal cognitive functioning based on the normative psychometric data.

### 2.4 MRI acquisition and analysis

MRI sequences were acquired on a 3-Tesla MRI scanner (Prisma Fit, Siemens Medical Solutions, Erlangen, Germany) with a 32-channel head/neck coil at the Functional Neuroimaging Unit of the CRIUGM, Montreal, Canada. High-resolution three-dimensional T1-weighted anatomical images were obtained using a volumetric magnetization-prepared rapid gradient echo (MP-RAGE; Mugler and Brookeman, 1991) sequence with the following parameters: repetition time (TR) = 2,400 ms, echo time (TE) = 2.17 ms, inversion time (TI) = 1,000 ms, flip angle = 8°, field of view (FOV) = 224 mm (read), FOV phase = 100%, voxel size = 1 × 1 × 1 mm^3^, slice thickness = 1 mm, slice and phase resolution = 100%, number of slices = 176, and echo spacing = 6.6 ms. The total scan time was approximately 6 min.

**Figure 1:**
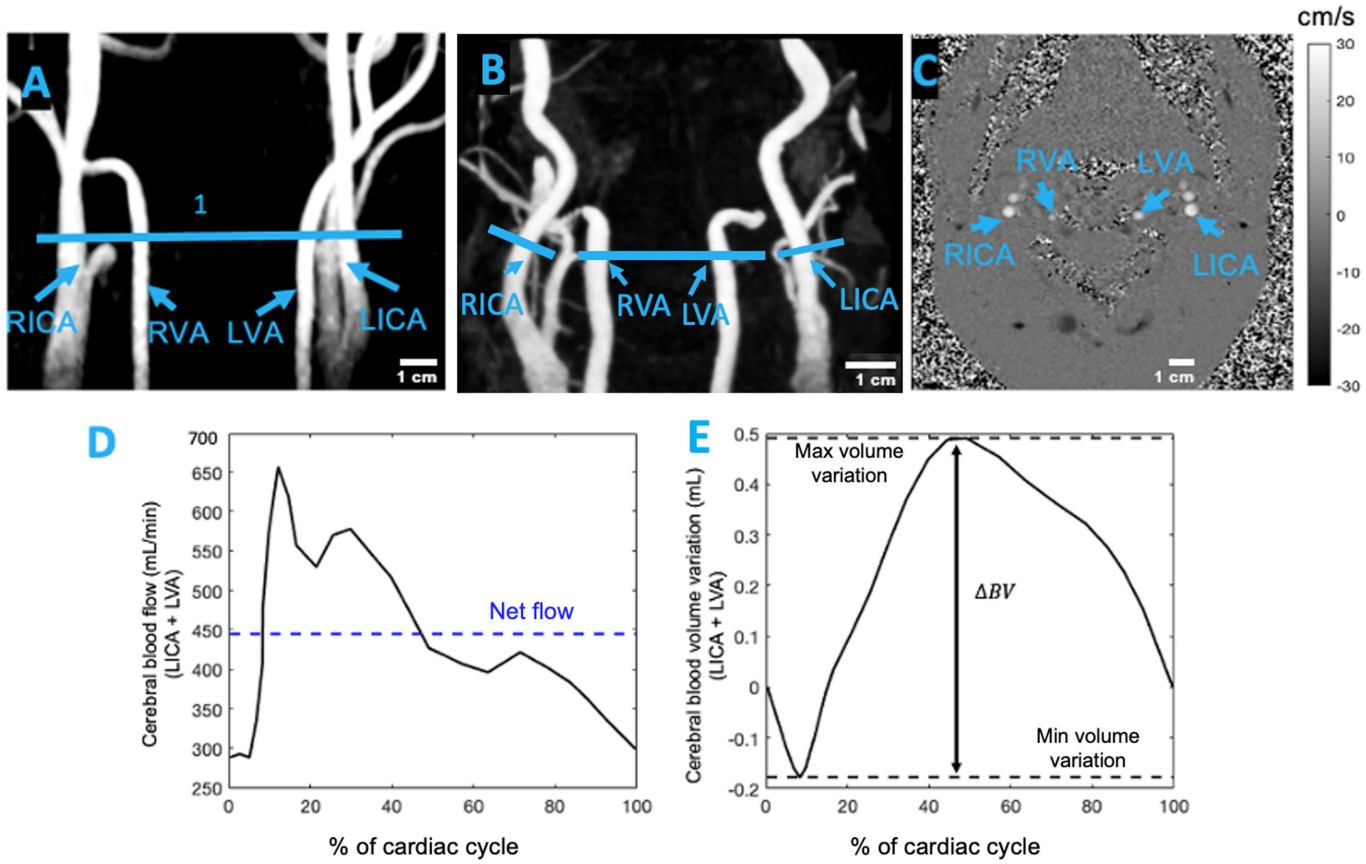
**(A)** Coronal view of 3D-TOF-MRA showing the neck arteries of a healthy young participant and **(B)** the neck arteries of a healthy older adult. The blue lines in **(A,B)** represent the location of the imaging plane between the C2 and C3 vertebrae. **(C)** Transverse slice of 2D PC-MRI showing the left internal carotid artery (LICA), right internal carotid artery (RICA), left vertebral artery (LVA), and right vertebral artery (RVA). This transverse plane for measuring pulsatility was selected between the C2 and C3 vertebrae, above the bifurcation of the common carotid artery. The grayscale bar indicates the average velocity (cm/s) in the ICAs and VAs at the end of systole (adapted from Mohammadi et al., 2021a). **(D)** Example of the cerebral blood flow waveform for the left hemisphere of a participant. ﻿**(E)** The cumulative integral of the cerebral blood flow was calculated after subtracting the net flow. The blood volume (BV) pulsatility index was determined as the difference between the maximum and minimum values.

Following the MP-RAGE sequence, two MRI sequences—3D time-of-flight magnetic resonance angiography (3D-TOF-MRA) and velocity-encoding phase-contrast scouts—were used to plan the PC-MRI. A 3D-TOF-MRA sequence with TR/TE = 20/3.11 ms, field of view (FOV) = 220 mm (read) and 75% (phase), slice thickness = 0.7 mm, voxel size = 0.6 × 0.6 × 0.7 FECAmm^3^, and flip angle = 15° provided images of the ICAs, VAs, and external carotid arteries in the neck. The 3D-TOF-MRA volumetric data were projected onto a 2D screen using the maximum intensity projection (MIP) algorithm in the Siemens software. These MIP images were used to select an acquisition plane between the C2 and C3 vertebrae, above the bifurcation of the internal and external carotid arteries, where cross-sections of the ICAs and VAs were clearly visible. The plane of acquisition was orthogonal to the artery, as indicated by a more circular outline in the artery cross-section (Figure 1C). Younger participants typically had straighter arteries (Figure 1A), which made selecting a perpendicular acquisition plane more time-efficient. In contrast, older adults often showed higher arterial curvature or tortuosity (Figure 1B), which complicated the selection of a perpendicular transverse plane. In such cases, an independent scan was required for each tortuous artery.

The selection of the acquisition plane was followed by a velocity-encoded phase-contrast (PC) scout sequence. This sequence acquired images at four velocity encodings (VENCs), specified by the operator. The selected VENC﻿ ranged from 50 to 90 cm/s. This scout sequence was used to identify a VENC that produced clear systolic and diastolic phase images without aliasing, and the acquisition time for the scout images ranged from 45 to 60 s. The chosen VENC was then used to perform a pulse plethysmography-gated two-dimensional PC-MRI sequence to measure velocity in the neck arteries. This sequence was adapted from Zarrinkoob et al. (2016) and had the following parameters: TR = 6.84–14.03 ms, TE = 8.16–8.51 ms, flip angle = 15°, FOV = 135 mm (read) and 100% (phase), slice thickness = 5 mm, voxel size = 1 × 1 × 5 mm^3^, reconstructed temporal resolution = 25 frames per cardiac cycle, and acquisition time = 5–7 min. During the PC-MRI session, participants were instructed to focus on a screen displaying a white cross on a black background and to concentrate on their breathing. All MRI data acquisition procedures were identical to those described by Mohammadi et al. (2021a).

The data collected from the PC-MRI were analyzed as follows. First, a polygonal region of interest (ROI) was defined in each frame for ICAs and VAs. Pixel intensities within the ROI were normalized to the maximum pixel value. An arterial mask for the phase image was created using a thresholding technique. Within the ROI, the background of the static tissue was identified using a method similar to that described by Pahlavian et al. (2021). Specifically, the background was defined as the region where the standard deviation was less than 15% of the standard deviation of the entire dataset. After identifying the static region, a horseshoe-shaped region was drawn, whose borders were chosen to balance the reduction in local phase errors that occur near the vessel lumen and the reduction in Gibbs ringing artifacts that occur farther from the vessel boundary. The mean velocity across the arterial cross-section was calculated and corrected by subtracting the mean velocity of the background region, as described by Wåhlin et al. (2010, 2012, 2014). Finally, the flow was determined as the product of the mean velocity and the arterial cross-sectional area for each frame.

Cerebral blood flow in each hemisphere was calculated as the sum of flow in both the ICA and VA over the cardiac cycle. The cumulative integral of the flow waveform, after subtracting the net flow, was used to compute the accumulated blood volume (Wåhlin et al., 2014). The difference between the maximum systolic volume and the minimum diastolic volume [maximum systolic volume – minimum diastolic volume] was defined as the cerebral blood volume (BV) pulsatility index per heartbeat, expressed in milliliters (Figures 1D,E; Wåhlin et al., 2014).

### 2.5 NIRS acquisition and analysis

#### 2.5.1 NIRS device and optode configuration

**Figure 2:**
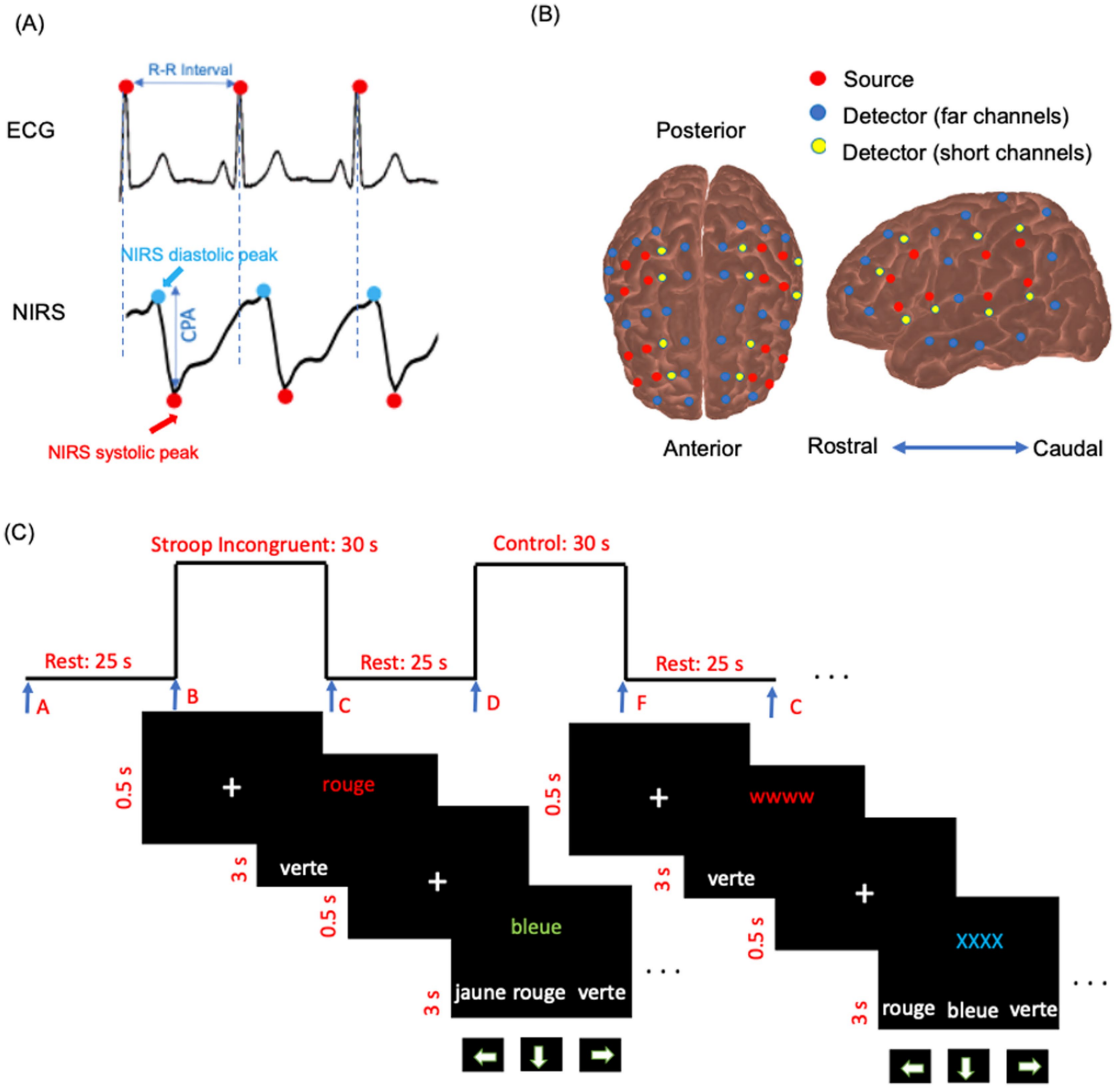
**(A)** Example of simultaneous ECG, and raw NIRS intensity data (830 nm) for a few seconds in a participant’s channel. The cerebral pulse amplitude (CPA) is defined as the amplitude from the systolic peak to the diastolic peak, baseline-corrected to the systolic peak in the NIRS data. **(B)** NIRS source and detector arrangement plotted on cortex. The arrangement is symmetric for the right and left hemispheres. Red circles indicate sources, blue circles represent far-channel detectors, and yellow circles denote short-channel detectors. **(C)** Schema of the color-word interference Stroop task diagram. Trials were displayed in French: *rouge* [red], *bleue* [blue], *jaune* [yellow], and *verte* [green].

Functional NIRS data were collected using a multichannel optical system (Brainsight, Rogue Research Inc., Montreal, Canada) with 695 nm and 830 nm monochromatic laser diodes at a 20 Hz sampling rate. The device included four bays, each equipped with four near-infrared sources, eight far-channel detectors, and four proximity detectors for short channels, resulting in 48 detectors, 16 sources, and 286 channels for both wavelengths. The sources and detectors symmetrically covered surface regions in both hemispheres, including regions or subregions of the upper and lower parietal areas; the upper, middle, and lower frontal areas (triangularis and opercularis); the precentral and postcentral regions; and the upper, middle, and inferior temporal areas (Figure 2B). A source–detector separation of 3–5.6 cm was used for the analysis. For short channels, the source–detector separation was 0.9 cm (Brigadoi and Cooper, 2015). The sources and detectors were mounted on flexible elastomer strips (Brainsight, Rogue Research Inc.) that were adjusted to fit the participant’s head according to the International 10–20 EEG System (Jasper, 1958). To minimize interference from ambient light, the experiment was conducted in a dark room with a lightweight black fabric covering the helmet for additional protection.

#### 2.5.2 Resting-state acquisition (NIRS)

Resting-state functional NIRS data were recorded simultaneously with the ECG for 8 min, consisting of eight blocks of 60 s each (Figure 2A). This resting-state paradigm followed an approach similar to that described by Eggebrecht et al. (2014). Participants were instructed to remain still and focus on their breathing. The pulsatile component of the resting-state NIRS data was used to extract cerebral pulse amplitude and heart rate (number of peaks in a given time). Following the method of Fabiani et al. (2014), only data from 830 nm were used to calculate cerebral pulse amplitude. As arterial blood is saturated with oxyhemoglobin, channels at the 830 nm wavelength are more sensitive to arterial pulsatility (Fabiani et al., 2014). During the resting state, with normal breathing, oxyhemoglobin concentration remains constant throughout the cardiac cycle. Other sources of physiological fluctuations that occur at lower frequencies, such as myogenic and neurogenic vascular activity and vascular endothelial function, are filtered out during the processing steps (see Section 2.8.1). Consequently, the primary mechanism modulating the NIRS signal is the change in regional arterial blood volume within the detector’s field of view (Mohammadi et al., 2021a). In the present study, NIRS data were used to extract cerebral pulsatile stress (see Section 2.8.1). According to the existing literature, pulsatile stress is defined as the product of peripheral pulse pressure and heart rate (Jae et al., 2012; Lima et al., 2021). In the context of the brain, absolute cerebral pulse pressure is not directly measured by NIRS; however, cerebral pulse amplitude serves as a proxy measure of cerebral pulse pressure. This approach is commonly adopted as cerebral pulse amplitude originates from the expansion of the arterial walls in response to local changes in pulse pressure, resulting in local changes in cerebral blood volume (Fabiani et al., 2014; Tan et al., 2017).

#### 2.5.3 Task-evoked hemodynamic acquisition (NIRS)

The resting-state acquisition was followed by a color–word matching Stroop task to study hemodynamic-evoked activations. This task was inspired by the paradigm described by Zysset et al. (2007). The task followed a block design format, consisting of eight blocks with a total duration of 540 s. Each block consisted of a 25-s resting period followed by a 35-s activation period (60 s per block). During each activation period, ten trials were displayed on the screen.

The task included two main experimental conditions: control (or neutral, NU) and Stroop interference (SI). During SI, the target stimulus was one of the four color words (green, blue, yellow, or red) displayed in incongruent ink colors on the upper half of the computer screen. On the bottom row, three color words (green, blue, yellow, or red) were presented in white ink as possible choices. Participants were instructed to select the ink color of the target word in the top row from the three options on the bottom row using the arrow keys on the keyboard. Of the eight task blocks, four were SI blocks, and the other four were NU blocks. The sequence of blocks (NU–SI–NU–SI–NU–SI–NU–SI) was presented in three different randomly intermixed orders across participants. Participants were given a maximum of 3 s to respond after each trial appeared on the screen. Before each trial, a white fixation cross (“+”) was displayed for 0.5 s to signal the start of the next trial (Methqal et al., 2017). The duration of each block, the resting period, and the response time were consistent across all task blocks. Participants were instructed to validate their responses if they answered faster than the allotted response time and then wait for the next trial (Figure 2C). In the NU condition, the target stimulus consisted of a color word (e.g., the word “blue”) printed in matching (congruent) ink color on the upper half of the screen. On the lower half, three color words (e.g., “blue,” “red,” “yellow”) were displayed in white ink as possible answers. Participants had to select, using the arrow keys, the option that corresponded to the ink color of the target stimulus. During the resting periods, participants were instructed to focus on their breathing and to avoid engaging in heavy mental activity. The resting-state NIRS acquisition (Section 2.5.2) always preceded the Stroop test. All participants underwent task training and began the experiment only after demonstrating a clear understanding of the task. Block triggers marking the start and end of each block were implemented using Expyriment 0.9.0 on Python 2.7. The schematic of the task paradigm is presented in Figure 2C.

### 2.6 Electrocardiogram and photoplethysmography recording and analysis

NIRS recordings were conducted simultaneously using ECG (BIOPAC BSL PRO MP36U-W, BIOPAC Systems, Inc., Goleta, CA, United States) with a sampling rate of 1,000 Hz (Figure 2A). ECG lead I signals were used for cardiac cycle gating of NIRS data. An R-wave detection algorithm was applied to calculate R–R intervals (MATLAB 2024a, The MathWorks, Inc., United States).

### 2.7 Tracing NIRS optodes and MRI co-registration

The locations of three fiducial landmarks—the left pre-auricular point (LPA), right pre-auricular point (RPA), and nasion—along with the positions of each NIRS source and detector, were digitized using a Polaris Vicra system (Northern Digital Inc., Ontario, Canada). The 3D coordinates of these sources and detectors were transformed into Montreal Neurological Institute (MNI) standard space. Each participant’s anatomical MRI was also normalized into MNI space. The transformed optode coordinates were then co-registered with the MRI data in MNI space and projected onto the cortical surface. Anatomical labels for each measurement channel were subsequently extracted using in-house scripts along with the AtlasViewer toolbox (Aasted et al., 2015).

### 2.8 NIRS data analysis

#### 2.8.1 Cerebral pulsatility index (NIRS-derived, resting state)

(1) *Preprocessing*: Saturated and noisy channels were manually identified and removed from further analysis. Periods with motion artifacts were also manually identified and removed from the analysis (Selb et al., 2015). (2) *Normalization*: The intensity data for each NIRS channel were normalized by dividing each channel’s data by its own mean (Tan et al., 2017). (3) *Filtering*: The data were subsequently filtered using a Butterworth filter with bandpass frequencies of 0.5–5 Hz to eliminate unwanted high-and low-frequency noise and to preserve the cardiac component (Afkhami et al., 2021). (4) *Scalp coupling index (SCI)*: To assess the quality of the heartbeats in each channel, the SCI metric was used, as described by Pollonini et al. (2014). Only NIRS channels with an SCI of ≥ 0.7 were used in the analysis (Mohammadi et al., 2021b). (5) *Separating heartbeat epochs and calculating beat-to-beat cerebral pulsatile stress*: ECG R–R intervals were used to separate heartbeat epochs. For each heartbeat epoch, the cerebral pulse amplitude was calculated, which was then multiplied by the heart rate (number of systolic peaks in 1 min) as follows: Heart rate×[(systolic peak−diastolic peak)/systolic peak ]. (6) *Quantile averaging*: For each channel, the obtained indices were divided into four quantiles. Only the second and third quantiles (representing the median values, more robust against noise) were averaged. (7) *Normalizing by the BV pulsatility index and obtaining the cerebral pulsatility index*: To account for individual variability in BV pulsatility that enters the brain, we intended to normalize the index obtained via quantile averaging, by dividing it by the BV pulsatility index (see Section 2.4). However, as our results indicated hemispheric differences in the BV pulsatility index (see Section 3.1), the normalization of NIRS channels for the left and right hemispheres was performed separately. This yielded the channel-wise cerebral pulsatility index. When averaged across all channels within each hemisphere, this index was referred to as the hemispheric cerebral pulsatility index, and when averaged across the cortex, it was referred to as the global cerebral pulsatility index.

#### 2.8.2 Hemodynamic-evoked activations for Stroop task (NIRS)

NIRS data from the Stroop task were processed using the HomER2 toolbox (Huppert et al., 2009), and the subsequent functional NIRS analysis was conducted following the methods of Hocke et al. (2018) and Aasted et al. (2016). (1) *Preprocessing*: Raw NIRS data were converted to optical density and visually reviewed, and saturated or noisy channels were identified and removed. (2) *Motion artifact correction*: Motion artifacts were identified and corrected using the “hmrMotionArtifact” function in HomER2 (tMotion = 0.5 s, tMask = 1 s, STDEVthresh = 15, AMPthresh = 0.5). The outcome was validated through visual inspection. Subsequently, channels with an SCI of ≥ 0.7 were retained. (3) *Short-channel regression*: Each far-channel signal was regressed against its corresponding short-channel signal. If short-channel data were unusable, the nearest adjacent short channel with the highest correlation with the target channel was used. (4) *Bandpass Butterworth filtering*: Bandpass Butterworth filtering was applied at the range of 0.01–0.2 Hz. (5) *Baseline correction*: Baseline correction was applied by subtracting the mean signal during a pretask resting period from the activation signal to ensure consistency in activation measures. (6) *Calculation of hemodynamic responses*: For each participant and NIRS channel, hemoglobin concentration changes were calculated using block averaging. Then, the average oxyhemoglobin concentration changes during the task condition were calculated and compared with the average during the resting condition (*baseline*). (7) *Within-group analysis*: One-sample *t*-tests were conducted to test whether hemodynamic responses differed significantly from baseline. (8) *Correction for multiple comparisons*: False discovery rate (FDR) correction was applied at the group level to account for multiple comparisons across channels (Benjamini and Hochberg, 1995). (9) *Group activation mask*: To calculate group activation mask, channels with significant oxyhemoglobin changes at the group level (after FDR correction, 𝑝 < 0.05) and that were consistently activated in at least 50% of participants were included. This mask was visualized by projecting significant channels onto the cortical atlas (Colin27 cortex; Aasted et al., 2015).

## 3 Results

### 3.1 Blood volume pulsatility index measured at the C2–C3 vertebral level (PC-MRI)

In older adults, within the left hemisphere, the BV pulsatility index was 0.691 ± 0.052 mL for the ICA and 0.301 ± 0.047 mL for the VA. In the right hemisphere, it was 0.670 ± 0.051 mL for the ICA and 0.291 ± 0.043 mL for the VA. Summing the contributions from the ICA and VA, the total hemispheric BV pulsatility index was 0.992 ± 0.070 mL in the left hemisphere and 0.960 ± 0.066 mL in the right hemisphere. A paired *t*-test revealed a statistically significant interhemispheric difference in the BV pulsatility index (*p* = 0.009).

In younger adults, the BV pulsatility index in the left hemisphere was 0.340 ± 0.028 mL for the ICA and 0.141 ± 0.016 mL for the VA. In the right hemisphere, it was 0.339 ± 0.017 mL for the ICA and 0.143 ± 0.027 mL for the VA. The total BV pulsatility index was similar between hemispheres, with the values of 0.481 ± 0.032 mL in the left hemisphere and 0.482 ± 0.0319 mL in the right hemisphere. A paired *t*-test showed no statistically significant interhemispheric difference in the BV pulsatility in younger adults (*p* > 0.05).

Regression analysis showed no significant effect of sex on the BV pulsatility index in younger adults (left hemisphere: *β* = −0.03, *p* = 0.22; right hemisphere: *β* = −0.02, *p* = 0.28) and older adults (left hemisphere: *β* = −0.07, *p* = 0.11; right hemisphere: *β* = −0.06, *p* = 0.13).

### 3.2 Functional NIRS data

#### 3.2.1 Reliability of the cerebral pulsatility index obtained from resting-state NIRS data

To assess the reliability of the cerebral pulsatility index, its consistency across eight resting-state blocks was evaluated using a correlation matrix (Tan et al., 2017). Pearson’s correlation coefficients were computed for all pairwise comparisons (28 in total), and their average was taken. The mean correlation coefficient (*r* = 0.897, 𝑝 < 0.001) showed a high level of consistency across blocks.

#### 3.2.2 Cerebral pulsatility index

**Figure 3:**
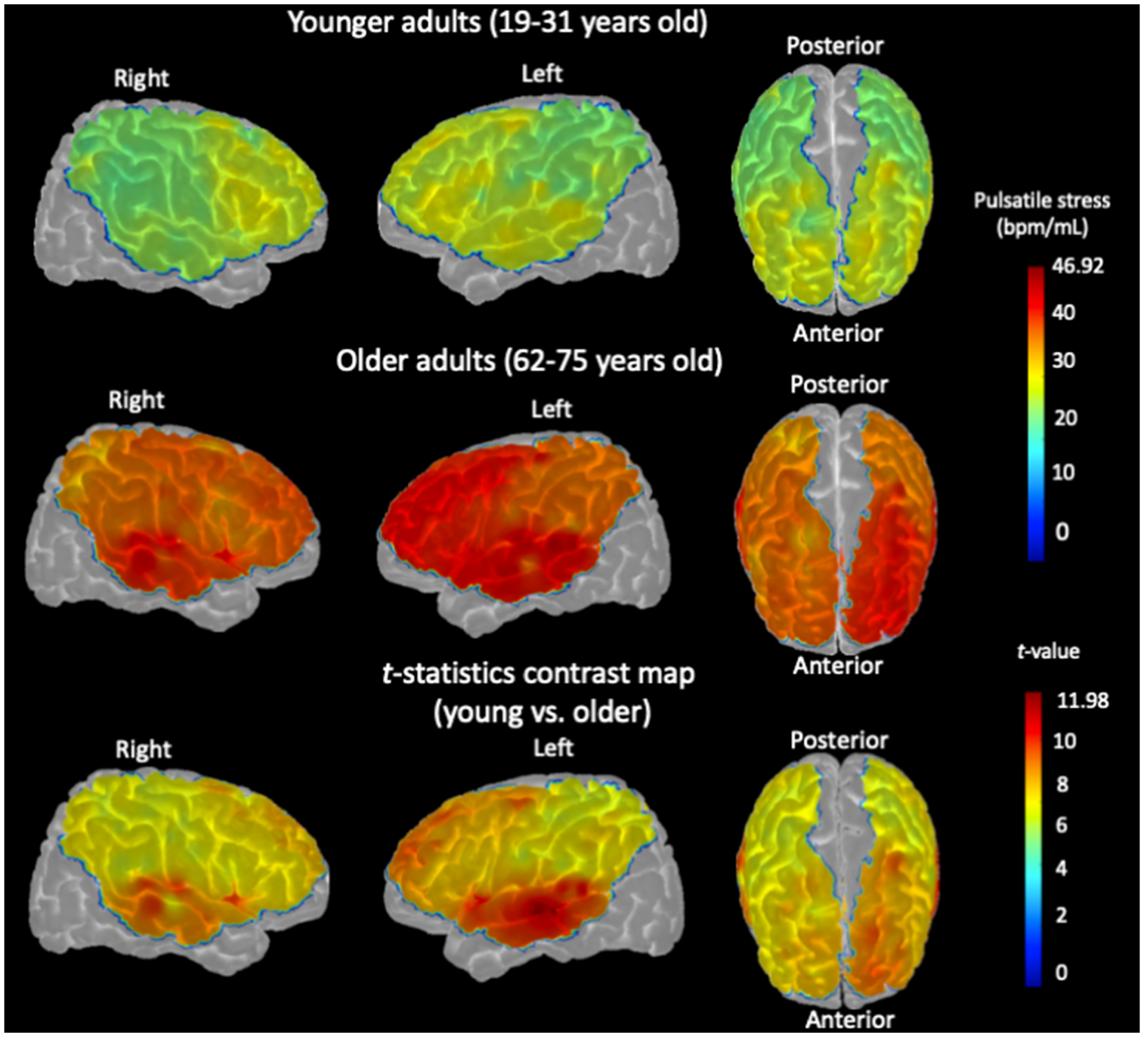
Channel-wise cerebral pulsatility index, averaged across all participants, projected onto the cortex of the Colin27 template. For each individual, the product of cerebral pulse amplitude and heart rate for each NIRS channel in each hemisphere was normalized by dividing by the hemispheric BV pulsatility index measured by PC-MRI. (Top row): Cerebral pulsatility index map for younger adults. (Middle row): Cerebral pulsatility index map for older adults. Higher values on the map (red regions) indicate greater pulsatility, whereas lower values (green regions) indicate a ﻿lower pulsatility index. (Bottom row): *t*-statistic contrast map of the channel-wise comparison between older adults and younger controls, with *p* < 0.05, FDR-corrected.

Channel-wise cortical projection of the cerebral pulsatility index across the cortex is represented in Figure 3, with younger adults qualitatively showing a lower cerebral pulsatility index, represented by green regions, and older adults showing a higher cerebral pulsatility index, indicated by red and yellow regions. In the middle row of Figure 3, older adults appear to have a higher cerebral pulsatility index in the left hemisphere (with more red regions) than in the right hemisphere (with more orange regions). To investigate this quantitatively, the hemispheric cerebral pulsatility index in the left hemisphere was 42.18 (±18.11) bpm/mL, but that in the right hemisphere was 38.12 (±16.17) bpm/mL. A paired *t*-test indicated that this difference was statistically significant in older adults (𝑝 < 0.001). In contrast, in younger adults, there was no significant difference between the left hemisphere and the right hemisphere [28.78 ± 17.88 bpm/mL and 27.65 ± 16.33 bpm/mL, respectively (𝑝 > 0.05)]. In addition, there was no association between the cerebral pulsatility index and sex for the right and left hemispheres in either group (𝑝 > 0.1).

Importantly, these NIRS findings were consistent with the PC-MRI measurements, which showed a similar pattern of hemispheric differences in the BV pulsatility index (see Section 3.1). First, the correlation between the BV pulsatility index (PC-MRI) and the hemispheric cerebral pulsatility index (NIRS) was calculated, which was significant in older adults (left hemisphere: *r* = 0.407, *p* = 0.025; right hemisphere: *r* = 0.397, *p* = 0.029). To confirm that this association was not an artifact of normalizing cerebral pulsatile stress to the BV pulsatility index (see Section 2.8.1), the raw correlation between this index and the BV pulsatility index (PC-MRI) was tested, which was also significant in both hemispheres for older adults (𝑝 < 0.05). However, in younger adults, the correlations were not significant for either right or left hemispheres (𝑝 > 0.1).

Furthermore, when comparing the cerebral pulsatility index between older and younger adults, the difference was statistically significant in both hemispheres, with a more pronounced effect in the left hemisphere (Figure 3, bottom row; two-sample *t*-test, 𝑝 < 0.001). All brain images were generated using the AtlasViewer toolbox, with the indices projected onto the Colin27 cortex (Aasted et al., 2015).

#### 3.2.3 Relationship between cerebral pulsatility index and pulse pressure

Pulse pressure was not significantly associated with either the hemispheric cerebral pulsatility index (left and right) or the global cerebral pulsatility index in younger or older adults (𝑝 > 0.1).

### 3.3 Stroop color–word interference and hemodynamic-evoked activations

**Figure 4:**
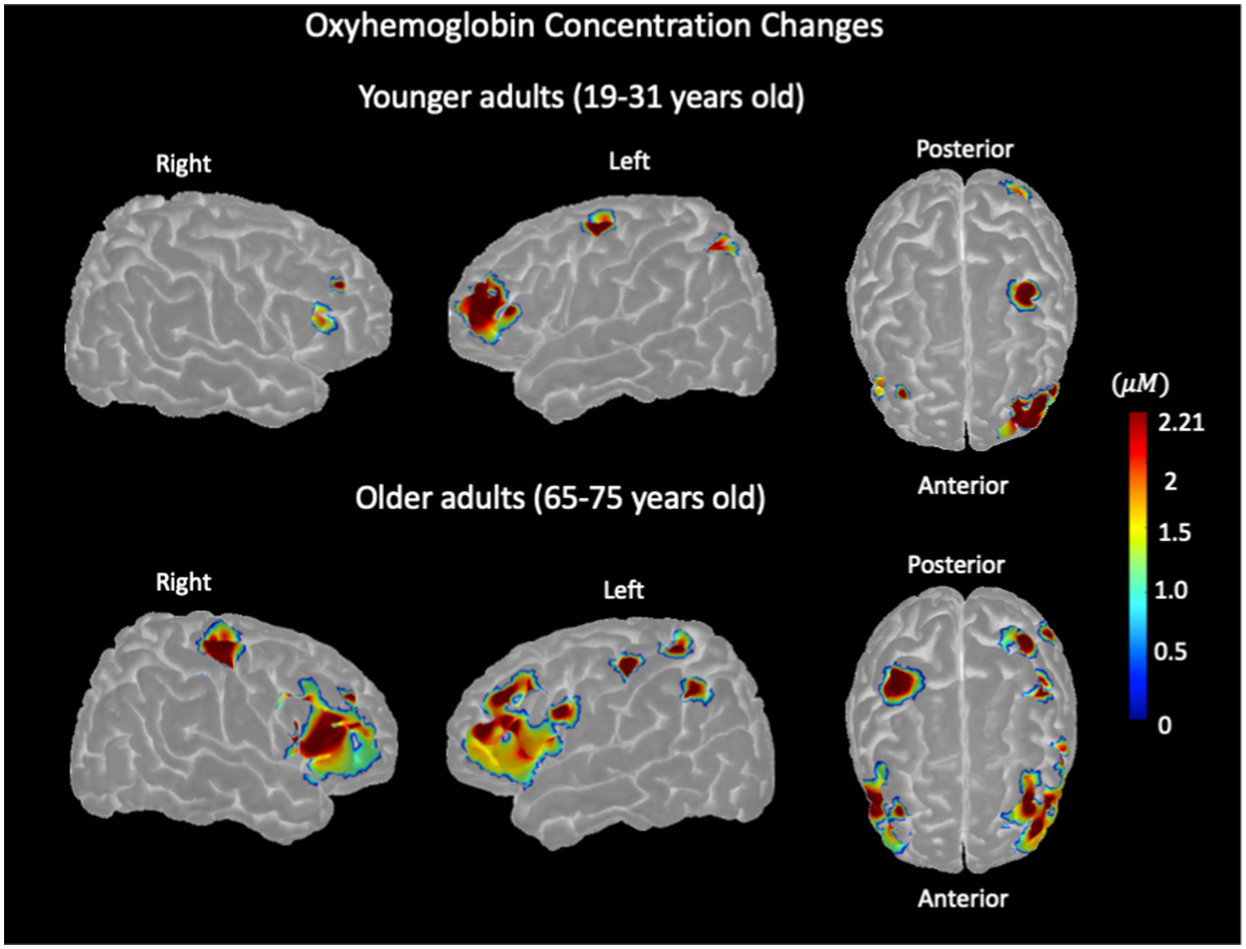
Stroop color–word interference task-evoked hemodynamic activation, representing oxyhemoglobin concentration changes measured with NIRS in younger adults (top row) and older adults (bottom row) for the left and right hemispheres.

The group-level activation map, within regions defined by the group mask, is represented in Figure 4, indicating oxyhemoglobin concentration changes of SI versus NU conditions in older and younger adults. In younger adults, significant task-evoked hemodynamic activation was predominantly observed in the left hemisphere subregions, especially in the superior frontal, middle frontal, inferior frontal (triangularis), and inferior parietal, with small activations in the inferior and middle frontal subregions of the right hemisphere. In older adults, significant activation was observed in left hemisphere subregions, including the middle frontal, inferior frontal (triangularis), precentral, postcentral, and inferior parietal subregions. In the right hemisphere of older adults, significant activation was observed in the middle frontal, inferior frontal (triangularis), and precentral subregions.

### 3.4 Local cerebral pulsatility index in the channels with hemodynamic-evoked activations and association between them

**Figure 5:**
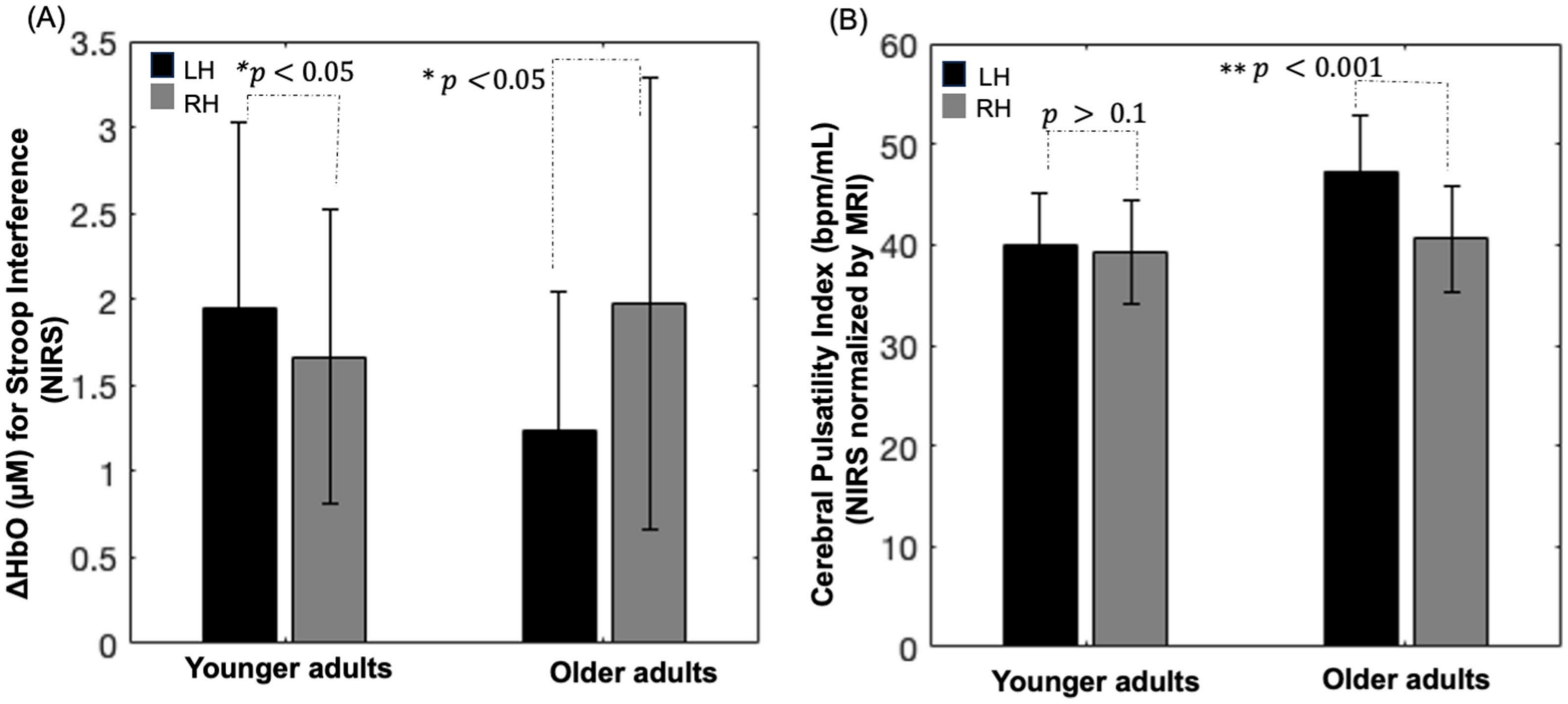
**(A)** Bar chart depicting oxyhemoglobin concentration changes within the group activation mask for the Stroop color–word interference task in each hemisphere. **(B)** Bar chart illustrating the cerebral pulsatility index in the corresponding channel. RH: right hemisphere; LH: left hemisphere; error bars: standard error.

As shown in Figure 5A (left), younger adults showed lower oxyhemoglobin concentration changes in the right hemisphere compared with the left (paired *t*-test, 𝑝 < 0.05), with no significant difference in the cerebral pulsatility index between the hemispheres (paired *t*-test, 𝑝 > 0.1; Figure 5B (left)). In older adults, oxyhemoglobin concentration changes were significantly lower in the left hemisphere and higher in the right hemisphere (paired *t*-test, 𝑝 < 05; Figure 5A (right)). In addition, their cerebral pulsatility index was significantly higher in the left hemisphere than in the right hemisphere (paired *t*-test, 𝑝 < 0.001; Figure 5B (right)).

**Figure 6:**
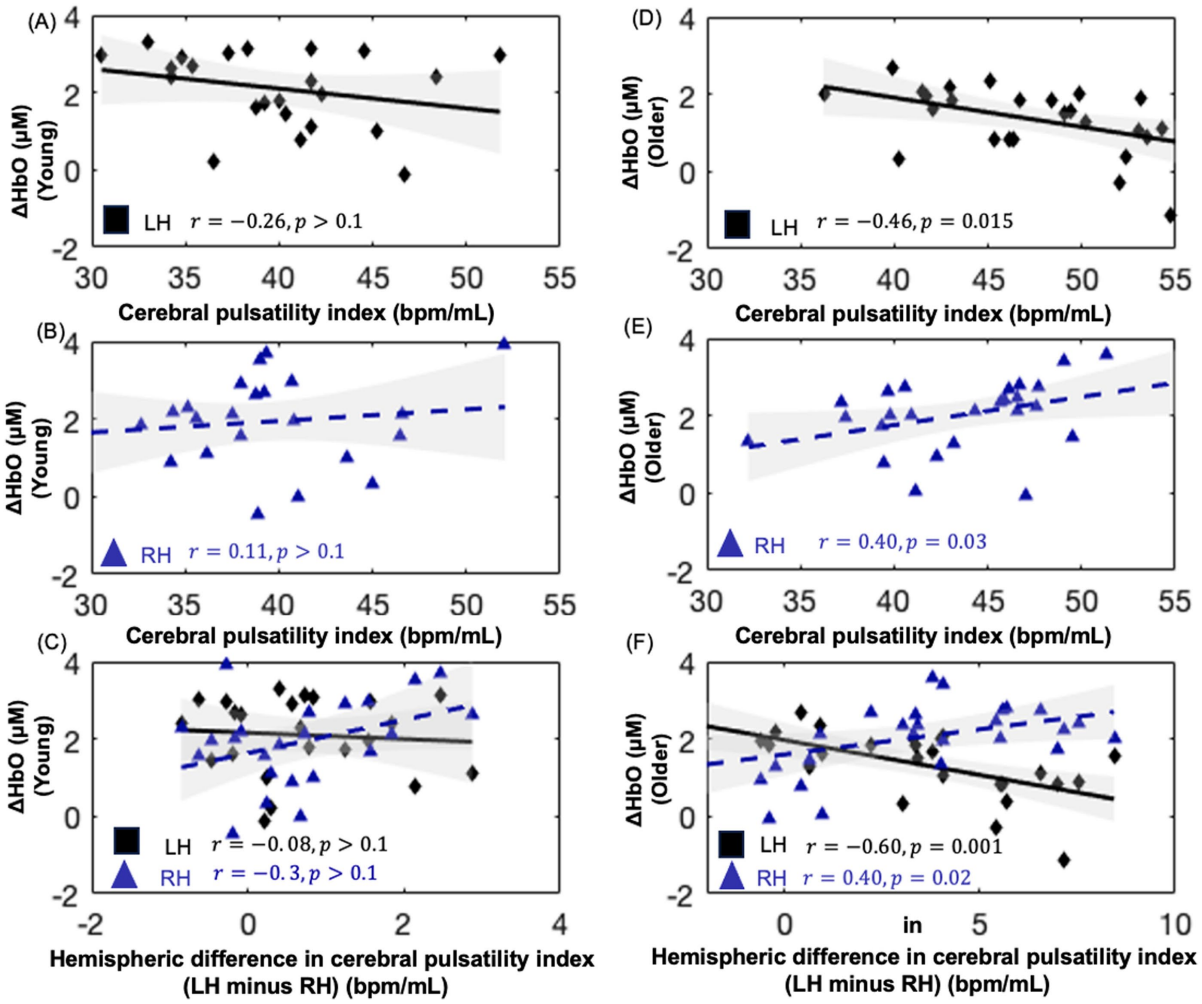
Scatter plots showing the relationship between oxyhemoglobin concentration changes and cerebral pulsatility index for NIRS channels within the hemodynamic activation mask of each group for the Stroop test. **(A,B)** depict this relationship in younger adults, while **(D,E)** depict this relationship in older adults. **(C,F)** present the interhemispheric difference in the cerebral pulsatility index (LH, left hemisphere minus; RH, right hemisphere) versus oxyhemoglobin concentration changes.

Notably, as shown in Figures 5A,B, visually inverse pattern was observed between oxyhemoglobin concentration changes and the cerebral pulsatility index in older adults, which prompted further investigation into the relationship between these two factors. Scatter plots showing this relationship in identical channels are presented in Figures 6A–F.

As shown in Figures 6D,E, in older adults, the cerebral pulsatility index in the left hemisphere was negatively associated with oxyhemoglobin concentration changes (*r* = −0.461, 𝑝 < 0.05). In contrast, in the right hemisphere, the cerebral pulsatility index was positively correlated with oxyhemoglobin concentration changes (*r* = 0.40, 𝑝 < 0.05). However, this association was not significant in younger adults (Figures 6A,B). In older adults, the interhemispheric difference in the cerebral pulsatility index was calculated by averaging cerebral pulsatility index values from channels in the group-level mask in the left hemisphere and subtracting this value from the corresponding average in the right hemisphere. Then, the interhemispheric difference in the cerebral pulsatility index was calculated for each participant, and its correlation with the average oxyhemoglobin concentration changes in the same channels from which the cerebral pulsatility indices were obtained﻿.

This analysis revealed that, in older adults, a higher interhemispheric difference in pulsatility was typically associated with decreased oxyhemoglobin concentration changes in the left hemisphere and increased oxyhemoglobin concentration changes in the right hemisphere (Figure 6F, 𝑝 < 0.05) for the color–word interference Stroop task. In younger adults, this association was not significant (Figure 6C; 𝑝 > 0.1).

### 3.5 Behavioral results of the Stroop test

Reaction times (RTs) and task performance were recorded during the SI and NU conditions alongside NIRS acquisition, and were analysed. In the SI condition, older adults had slower RTs (1181.26 ± 217.22 ms) than younger adults (906.16 ± 181.97 ms). In the NU condition, older adults averaged 916.47 ± 97.11 ms, whereas younger adults averaged 778.76 ± 89.11 ms.

Task performance and error rate were calculated as follows: (number of trials with correct answers/total number of trials) × 100 and (number of trials with incorrect answers/total number of trials) × 100, respectively. Trials with no response were excluded from both performance and error rate calculations. Older adults had a performance of 81.7% (± 12.21) and 87.1% (± 4.6), whereas younger adults scored 95.7% (± 3.18) and 98.9% (± 1.18) in the SI and NU conditions, respectively. The error rate for older adults was 14.73% (± 5.6) and 5.96% (± 1.6) in the SI and NU conditions, respectively. Younger adults showed error rates of 3.75% (± 0.16) and 1.96% (± 0.14) in the SI and NU conditions, respectively. A repeated-measures ANOVA was carried out to examine the effects of condition (SI vs. NU) as a within-subject factor and age (younger vs. older) as a between-subject factor on RT and error rate. Prior to the analysis, the assumptions of normality (Shapiro–Wilk test, *p* > 0.05) and homogeneity of variances (Levene’s test, *p* > 0.05) were verified.

For RT, significant main effects were observed for both condition [*F* (1, 58) = 53.658, 𝑝 < 0.001] and age [*F* (1, 58) = 379.85, 𝑝 < 0.001]. This indicates that RTs were significantly slower in the SI condition than in the NU condition and that older adults responded more slowly overall than younger adults. In addition, there was a significant interaction between age and condition [*F* (1, 58) = 7.359, 𝑝 = 0.008]. This suggests that the difference in RT between conditions was more pronounced in older adults, indicating that they were more affected by the increased cognitive demands of the SI.

For error rate, significant main effects were observed for both condition [*F* (1, 58) = 44.822, *p* < 0.001], and age [*F* (1, 58) = 130.17, *p* < 0.001]. This indicates that participants made more errors in the SI condition than in the NU condition and that older adults showed higher overall error rates. A significant interaction between age and condition was also observed [*F* (1, 58) = 7.439, *p =* 0.008]. This suggests that older adults showed a greater increase in error rates from the NU to the SI condition than younger adults.

## 4 Discussion

This study showed a significant interhemispheric difference in the cerebral pulsatility index in older adults, with consistently higher values in the left hemisphere than in the right hemisphere. This pattern was evident in both the BV pulsatility index measured by PC-MRI and the cerebral pulsatility index assessed by NIRS by NIRS. However, such interhemispheric differences were not observed in younger individuals. In addition, within the NIRS channels that showed hemodynamic-evoked activations for SI versus NU conditions, older adults showed a higher cerebral pulsatility index than younger adults. Furthermore, as the interhemispheric pulsatility difference increased in older adults, with a higher index in the left hemisphere than in the right hemisphere, oxyhemoglobin concentration changes decreased in the left hemisphere and increased in the right hemisphere. These findings highlight age-related differences in the cerebral pulsatility index and its potential impact on task-evoked hemodynamic responses.

### 4.1 Cerebral pulsatility index and blood volume pulsatility index

The optical index of cerebral pulsatility encompasses two key variables: local cerebral pulse amplitude, which serves as a proxy for local pulse pressure (Tan et al., 2017), and heart rate. Local pulse pressure can vary regionally, with different brain areas experiencing varying levels of pulsatility and thus differing mechanical stresses on the microcirculation (Kim et al., 2021). This variation occurs because each heartbeat generates a pressure gradient that propagates through the arterial tree. Differences in vascular structure and the presence of arterial damage in different arteries lead to variations in local wall expansion (Kim et al., 2021). This expansion is also influenced by local arterial stiffness, which in turn affects the volume of oxygenated blood that can be accommodated. Changes in the volume of oxygenated blood can be measured by variations in the intensity of the NIRS signal (Windkessel model; Mohammadi et al., 2021b). On the other hand, heart rate determines how frequently tissues are exposed to pulsatility and is influenced by factors such as age, autonomic function, cardiovascular health, and physical fitness (Jae et al., 2012). By combining cerebral pulse amplitude and heart rate, it is possible to gain a more comprehensive understanding of the mechanical stress imposed on the tissue.

Importantly, findings of this study revealed a significant association between the BV pulsatility index and the cerebral pulsatility index. It is crucial to emphasize that BV pulsatility is measured in the arteries using PC-MRI (Wåhlin et al., 2014), whereas pulsatility within the cerebral microcirculation is measured using NIRS (Mohammadi et al., 2021b), which consists of arterioles and potentially capillaries and veins (Mitchell, 2008). Both indices are related to blood volume but at different levels: BV pulsatility reflects the volume of blood entering the brain with each cardiac cycle, whereas NIRS-derived pulsatility reflects beat-to-beat local changes in arterial blood volume within the local cerebral microcirculation, specifically in the region probed by the NIRS source and detector. To account for interindividual differences in blood volume input, cerebral pulsatile stress was normalized by dividing it by BV pulsatility. This approach helps account for variations in systemic blood volume delivery, allowing for a more precise assessment of local microvascular pulsatility. Furthermore, this may provide insights into the vascular health of the intermediate arterial pathways. Hypothetically, discrepancies between these two measures could reflect differences in arterial compliance, resistance, or ability to dampen pulsatile energy as blood travels from large arteries to the microvascular bed, and these discrepancies could serve as a potential biomarker of cerebrovascular health. Indeed, the literature suggests that aging is associated with increased arterial stiffness, increased pulsatility, reduced vascular compliance, higher endothelial dysfunction, and diminished vascular reactivity, collectively indicating both mechanical and physiological stress on the cerebrovasculature (Mitchell, 2008; Joannidès et al., 2014; Iulita et al., 2018). Hence, these age-related changes may impair the ability of local arteries to sufficiently expand and accommodate the surge of oxygenated blood necessary for effective neurovascular coupling (Mohammadi et al., 2021a).

Furthermore, the findings of this study indicate that pulse pressure, a peripheral index of pulsatility, was not significantly associated with the cerebral pulsatility index in either younger or older adults. This may suggest that cerebral pulsatility is influenced by other regulatory mechanisms such as cerebrovascular tone control and baroreflex function, which modulate the blood flow inside the brain (Ogoh and Tarumi, 2019). Therefore, cerebral pulsatility should be measured directly in the brain for a more accurate assessment.

### 4.2 Interhemispheric difference in the cerebral pulsatility index

Older adults showed an interhemispheric difference in the pulsatility index, with a higher average index in the left hemisphere than in the right hemisphere. This finding is consistent with those of previous research showing a higher pulsatility index in the left insula than in the right insula (Atwi et al., 2020). This difference may be attributable to anatomical variations in arterial branching from the central arteries to the cerebral circulation, which could lead to differences in pulsatility. Specifically, the right ICA branches from the right common carotid artery, which branches off the right brachiocephalic artery, whereas the left ICA originates directly from the left common carotid artery, which stems from the aortic arch (Nguyen and Duong, 2020).

These structural differences suggest that age-related increases in aortic stiffness and central arterial pulsatility may have a more pronounced impact on vascular health in the left hemisphere than in the right hemisphere. To test this hypothesis, future studies should incorporate TOF imaging of arteries from the heart to the brain, along with pulsatility measurements in each artery, to better understand how central arterial pulsatility propagates to the cerebral vasculature. Notably, in younger adults, the difference in pulsatility between the left and right hemispheres did not reach statistical significance, which suggests that the interhemispheric difference in the cerebral pulsatility index observed in older adults is primarily related to aging.

### 4.3 Local cerebral pulsatility index and local hemodynamic-evoked activations

The findings of this study indicate that in older adults, a higher cerebral pulsatility index is negatively associated with task-evoked oxyhemoglobin concentration changes in the left hemisphere (Figure 6D) and positively associated with oxyhemoglobin concentration changes in the right hemisphere (Figure 6E). A similar but nonsignificant trend was observed in younger adults (Figures 6A,B).

The association between local pulsatility and local hemodynamic response suggests that increased pulsatility may impair the ability of the local vasculature to effectively regulate the blood flow and pressure. In particular, the left hemisphere, due to its higher pulsatility, may be more functionally impaired, potentially leading to a greater reduction in neurovascular coupling efficiency. This hypothesis could explain why tasks that predominantly engage the left hemisphere in youth may experience reduced neurovascular resources with aging, likely due to chronic exposure to vascular stress. However, the differing behavioral responses between the right and left hemispheres remain difficult to explain comprehensively. One possible factor is the lower average pulsatility observed in the right hemisphere, as shown in our results, which may reflect greater neurovascular coupling resources than in the left hemisphere. This suggests that, in response to age-related increases in local stress, the brain may engage neural mechanisms by recruiting regions with lower stress, possibly those with higher neurovascular coupling capacity. We hypothesize that this age-related adaptation may manifest as increased engagement of the right hemisphere with aging, functional reorganization (Zysset et al., 2007; Langenecker et al., 2004), or an age-related shift in neuroactivation (Methqal et al., 2017), as observed in several studies. This finding is in agreement with the findings of Cabeza (2002) during a verbal working memory task, as well as our results presented in Figure 5, which show higher left hemisphere activation in younger adults and a shift toward higher right hemisphere activation in older adults.

Stern (2009) discussed how aging disrupts typically activated brain networks, leading to processing inefficiencies. As an adaptation, the aging brain recruits additional neural resources, engaging alternative networks that are less affected by aging-related disruptions. This process, known as neurofunctional reorganization, is considered an attempt by the aging brain to maintain performance (Ansado et al., 2013). While Stern did not specifically link these inefficiencies to pulsatility, his thought framework allows for the possibility that age-related variations in regional cerebral pulsatility—causing higher local mechanical and physiological stress in some regions more than other regions—may contribute to neurovascular disruption in some regions more than others. Accordingly, we suggest that alternatively recruited regions may show better vascular health, as indicated by lower regional cerebral pulsatility.

In terms of activation patterns, the older group showed more widespread activation across both hemispheres, particularly in prefrontal regions, whereas younger adults showed more focused activation in the left prefrontal and parietal areas (Figure 4). This pattern is consistent with the literature reporting predominantly right hemisphere dominance during successful inhibition in older adults (Garavan et al., 1999). The hypothesis regarding the relationship between cerebral pulsatility and brain activation in cognitive tasks may also explain the differences in activation patterns between older and younger adults. Hypothetically, age-related higher pulsatility in the left hemisphere could induce broader activations in the adjacent regions as the brain seeks to address limitations in its typically involved processing pathways. Although this mechanism may not be entirely sufficient, its conjunction with the increased engagement of the right hemisphere in the broader pattern may explain why older adults show more diffuse and widespread activation patterns (as opposed to the more localized activation observed in younger adults). To investigate this, we would require a denser NIRS optode arrangement that would allow us to differentiate between hemodynamic activations in adjacent brain areas, which is beyond the scope of this study.

Notably, two important points should be considered. First, while examining the relationship between pulsatility and hemodynamic-evoked activations, it is essential to distinguish between the rapid response of cerebral vessels to cardiac-induced pulsatility (reflected in the cerebral pulsatility index, approximately 1 s) and the slower response driven by neural activation (reflected in neurovascular coupling, approximately 4–6 s). Here, we emphasize that pulsatility (a fast response) and neurovascular coupling (a slower response) are distinct physiological phenomena operating on different timescales, yet they are associated, as shown in Figure 6. Second, the brain undergoes various forms of functional reorganization to maintain cognitive abilities during aging, and these changes are unlikely to be universally predictable. Therefore, the relationship between vascular health and functional reorganization, including the role of cerebral pulsatility, is only one of many factors influencing brain reorganization in aging.

Regarding the performance of the Stroop test, our findings indicate that older adults showed significantly slower RTs and higher error rates than younger adults in both Stroop conditions, with a more pronounced performance decline in the SI condition. This age-related decline in cognitive performance is consistent with the findings of previous research showing that increased cognitive demands affect older individuals due to possibly reduced processing efficiency and executive function capacity (Gajewski et al., 2020). Furthermore, the significant interaction between age and condition for both RT and error rate suggests that older adults experienced greater difficulty adapting to the increased cognitive demands of the SI task.

### 4.4 Limitations

Although the findings of this study support the underlying mechanisms of cognitive aging, their interpretation is limited by several issues. (1) Our interpretation is consistent with the biomechanical hypothesis linking cerebral pulsatility to age-related hemodynamic-evoked activations; however, a direct cause-and-effect relationship between these two parameters could not be established in this study. (2) Our results were specific to cerebral pulsatility and the Stroop color–word interference task. These results should be validated using other cognitive tasks with varying levels of difficulty to ensure generalizability. (3) Although we specifically included participants without significant risk factors to minimize confounding variables, we did not assess markers for neurodegenerative diseases. This limits our ability to account for the potential impact of neurodegenerative diseases that may influence cognitive test performance and measures of pulsatility. (4) This study had a relatively small sample size, and further studies with larger sample sizes are needed to confirm and build upon these findings.

## 5 Conclusion

In conclusion, our findings suggest that cerebral pulsatility is a correlate of hemodynamic-evoked activations for the Stroop test in older adults. Specifically, aging is characterized by increased interhemispheric differences in pulsatility, with the left hemisphere showing higher pulsatility than the right hemisphere in older adults, an observation not seen in the younger group. This higher pulsatility in the left hemisphere was negatively correlated with oxyhemoglobin concentration changes in the left hemisphere and positively correlated with those in the right hemisphere in older adults. The significance of these findings lies in the potential of cerebral pulsatility as a contributor to age-related functional adaptations and underscores the importance of further research to better understand the role of cerebral pulsatility in adaptive brain aging.

## Data Availability

The data and code supporting the findings of this study are available from the corresponding author upon reasonable request. A limited version of the dataset and analysis scripts can be shared in accordance with institutional and ethical guidelines.
